# Adolescent Lifestyle and Behaviour: A Survey from a Developing Country

**DOI:** 10.1371/journal.pone.0012914

**Published:** 2010-09-27

**Authors:** Waris Qidwai, Sidra Ishaque, Sabeen Shah, Maheen Rahim

**Affiliations:** 1 Department of Family Medicine, Aga Khan University, Karachi, Pakistan; 2 Medical College, Aga Khan University, Karachi, Pakistan; Penn State University, United States of America

## Abstract

**Introduction:**

Adolescents form two-thirds of our population. This is a unique group of people with special needs. Our survey aims to identify the lifestyle and behavioral patterns in this group of people and subsequently come up with issues that warrant special attention.

**Methods:**

A survey was performed in various schools of Karachi. Data collection was done via a face-to-face interview based on a structured, pre-tested questionnaire. Participants included all willing persons between 12–19 years of age.

**Results:**

Most adolescents with lifestyle issues fell in the age group of 16–18 years. Females were more depressed than males and had more sleep problems. Substance abuse and other addictions were documented more in males. Watching television or listening to music was stated as the most common late night activity (61.8%) and therefore was also referred to as the contributory factor for less than eight hours of sleep each day. (58.9%) of the respondents are getting less than eight hours of sleep daily. (41.5%) of the respondents who felt depressed sought treatment for it. Quite a few of them were also indulged in substance abuse and other addictions. Only (16.8%) of the respondents opined that physical activity is essential for health. Thirty-five adolescents out of all the respondents were smoking cigarettes currently, whereas 7% of the respondents chewed paan (areca nut). Peer pressure was the most common reason (37.1%) to start smoking.

**Conclusion:**

Adolescents need to be treated as a distinct segment of our population and it is important to realize and address their health and lifestyle problems. Inadequate sleep, depression and smoking were the leading unhealthy behaviours among the respondents. Families can play an important role to help these adolescents live a healthier life. Further research studies should be carried out to highlight issues of concern and their possible solutions in this population.

## Introduction

Adolescent population and health of adolescents is a very special issue and is focus of attention globally for various reasons. The world today is home to the largest generation of 10–19 year olds in our history and number over one billion and their population is continuously increasing.

The demands on young people are new and unprecedented; their parents could not have predicted many of the pressures they face. How we help adolescents meet these demands and equip them with the kind of education, skills, and outlook they will need in a changing environment will depend on how well we understand their world.

The first step toward deepening our understanding is to clarify the concept of adolescence. There is no universal method for doing so, and in Pakistan policies and programs affecting young people are bound to be affected by a lack of consistency. “Adolescents” and its cognates are variously defined. The lines between childhood, adolescence, and adulthood may differ by culture and region. The CDC uses the terms “adolescents and young adults” for those aged 10 to 24, inclusive, [1] usually broken into 3 age groups (ages 10–14, 15–19, and 20–24).

The population aged 15–24 in Pakistan was estimated to be approximately 27 million in 2000, and it is expected to continue to increase, reaching 44.6 million in 2020. This is an increase of 39 percent in just 20 years. This age group accounts for almost one quarter of the population in Pakistan and the peak number of youth will be reached in the year 2035 [2].

Basic data on education, employment, and reproductive health among adolescents shows that they are not receiving the adequate schooling and capability building to equip them for the future. It also follows from the Mensch et al. characterization of adolescence that the period of transition to adulthood must equip young people with the education, skills, decision-making power, and information to function as responsible adults in society.

Adolescents are a unique population with specific health concerns and needs. Adolescence is the peak age of onset for serious mental illness like depression and psychosis. Over load of stress from physical, emotional, social and sexual change makes adolescents overloaded with stress which can result in anxiety, withdrawal, aggression, poor coping skills and actual physical illness.

The adolescent period is characterized by its rapid physical and psychological changes in the individual, together with increasing demands from and influence of peers, school and wider society. It is well documented that behaviors developed during this period influence health in adulthood [2]. Several health compromising behaviours (e.g. smoking, alcohol) as well as health enhancing behaviours (e.g. physical exercise) are adopted in adolescence and they often persist into adulthood [3]. The World Health Organization estimates that 70% of premature deaths among adults are due to behavior (smoking, illicit drug use, reckless driving) initiated during adolescence [4]. Therefore, helping adolescents establish healthy lifestyles and avoid developing health risk behaviors is crucial and should be started before these behaviors are firmly established.

Adolescence is characterised by a strong tendency to experiment with risk behaviour. The desire for novelty and the courage for experiment are much greater in adolescence than in later life [5]. Most commonly reported behaviors in this population include such as watching TV, playing video games, hitting others, smoking and drinking alcohol, as lack of sleep, swearing, throwing things, and vandalism [6],[7],[8].

In recent years there has been considerable research interest in determining the prevalence of self-harm among adolescents [9]. This is not surprising as self-harm is a key predictor of completed suicide [10].

Furthermore, considerable gender differences can be found with relation to health-related behaviour, both in adults and in adolescents. Generally, males exhibit more health-risk and less health-protective behaviour than females [11],[12]. However, in recent years some studies have reported a remarkable increase in smoking among women [13],[14].

With growing evidence that chronic inadequate sleep results in negative daytime consequences (e.g., daytime sleepiness, depressed mood) [15],[16] interventions designed to reverse adolescent delayed sleep timing may help alleviate these problems [17].

The concept of adolescence as a distinct period of human development is still fairly new in Pakistan. The Pakistan population Association reports that 65% of Pakistani households contain one or more adolescents[18]. Adolescence often turns away from parents and health care providers towards peers for support and guidance. Nonetheless, a brief look at the available information is therefore important in our pursuit of identifying and highlighting the health, lifestyle and behavioural issues and recommends possible ways to deal with them, so as to promote a healthier lifestyle in this population. The purpose of the study was to elucidate the lifestyle and behavior of adolescents in the country and identify the major health risk behaviours in this age group.

## Materials and Methods

### Sample and settings

A cross sectional study was carried out using a questionnaire based interview. It was conducted in the local schools of Karachi. Karachi is the largest city of Pakistan comprising of mixture of all major ethnic groups (Punjabi, Sindhi, Mohajir, Baloch, Pathan, Memon, and Kashmiri). These schools were co education, English medium.

We approached all subjects aged 12 to 18 years. Subjects with known psychiatric disorder (as reported by the teacher) were excluded from the study.

Individual schools and classes were selected randomly; completed the questionnaire in their classrooms at school under the guidance of the interviewers and the teachers; data collection was continued for four weeks during the month of April 2009.

With a rejection rate of 8.1% we managed to complete 401 questionnaires. Commonest reason for refusal was ‘feeling unwell’. Interviews were conducted by doctors, who were also involved in designing the questionnaire. The time period for data collection was one month (March 2009-April 2009).

### Included variables and definitions

The common health and lifestyle behaviours were defined as follows:

- Physical exercise. Organized physical exercise is obtained by summarizing, for each respondent, the total frequencies of participating in exercise organized by (a) schools or workplaces (physical training lessons were excluded), (b) sports clubs and (c) other associations or clubs. Classification is: daily, weekly (at least thrice a week, for more than an hour).
- Internet use per week: <20 hours per week, >20–40 hours per week, >40 hours per week.
- Smoking: never tried, experimental or occasional (have smoked at most 50 times, but do not smoke daily), 1–5 cigarettes a day, 6–19 cigarettes a day, 20+ cigarettes a day.
- ‘Often’ feel depressed/anorexic: the term “often” included (1) at least twice every week.
- Bedtime:/Sleep: regular, irregular. (Before mid-night, after mid-night, less than eight hours).

### Questionnaire

**a. Development.** The initial questionnaire was developed based on the prior experience of investigators, input from colleagues, peers as well as patients. The draft so prepared was then pre-tested on 25 respondents and no changes were deemed necessary to be made in the questionnaire based on this pre-testing. A meeting of the investigators was held prior to the administration of the questionnaire in order to maintain uniformity in its administration; hence reducing chances of interviewer's bias in the study.

**b. Sections.** The questionnaire covered demographic characteristics, common health issues (acne, impaired vision, dental caries, hair fall, headache, mood changes), lifestyle and habits (frequency and timing of meals, daily water intake, sleep hygiene, late night activities, music preferences, internet use, regular exercise, leisure activities, smoking, recreational drug use). These variables were selected based on previous studies where they are the most commonly reported in this age group [11].

The last part assessed depression using DSM 4 criteria [19]. It consisted of questions regarding mood changes, lack of interest in daily activities, weight loss or gain, and sleep pattern, guilt, and suicidal ideations. We used cut off value of more than or equal to 5 for identifying depression. Individuals who were screened to have depression were counseled and advised to seek help from a psychiatrist.

### Statistical Analysis

Data was entered and validated using Epi-data version 3.1. It was cleaned for invalid and out of range values, missing values and duplicate ID numbers. The data was then imported to Windows Statistical Package for Social Sciences (SPSS) version 16.0 using DBMS [20]. The data was analyzed using Windows SPSS version 16.0.

Descriptive statistics included frequencies for continuous and categorical variables.

### Ethical Consideration

Written informed consent was obtained from the subjects after explaining the study objectives. The subjects were free to withdraw at any time without giving any reason. Strict confidentiality was maintained throughout the process of data collection, entry and analysis. All efforts were made in this study to fulfill the ethical considerations in accordance with the ‘Ethical principles for medical research involving human subjects’ of Helsinki Declaration [21].

## Results

**Figure 1:**
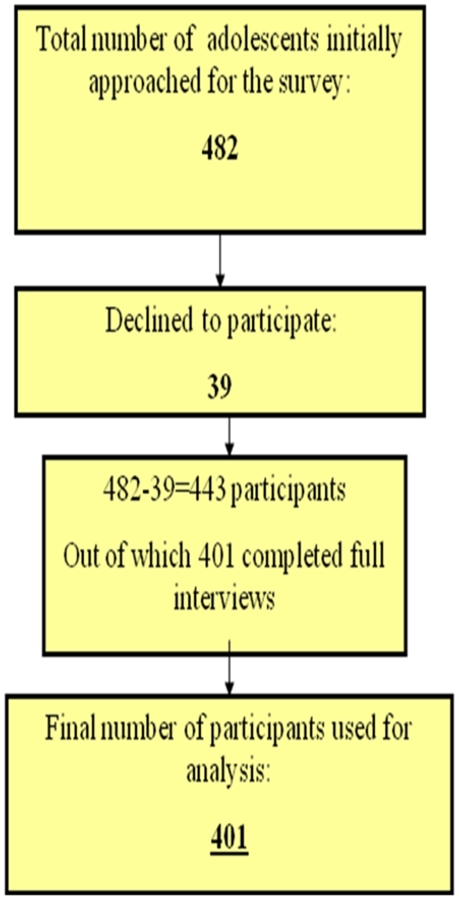
Selection of respondents finally participating in the study.

A total of 482 individuals were approached for this survey, 39/482(8.1%) declined to participate; therefore the response rate for the study was 91.9%. Out of a 443 respondents who participated in the study, 401 respondents completed full interviews which were used for primary analysis. **Figure 1** details the total of number of respondents who finally participated in the study:

56.4% of the respondents were males. The mean age of the respondents was 14.39 years. The majority (99.5%) of the respondents were single. Urdu was the most commonly spoken language (32.4%), followed closely by Sindhi and Punjabi. The socio-demographic characteristics of our study population (n = 401) are given in **Table S1**.

More than half of the respondents (63%) suffered from hair loss. This was followed closely by respondents with dental caries.(50.8%). Regarding the eating habits, the majority stated that they ate three meals and five to eight glasses on an average each day and considered their eating habits as being ‘just right.’ Watching television or listening to music was stated as the most common late night activity (61.8%) and therefore was also referred to as the contributory factor for less than eight hours of sleep each day. Interesting associations were drawn with regards to the practices of the individuals who were depressed. A large percentage (41.5%) of the respondents who were depressed sought treatment for it. Quite a few of them were also indulged in substance abuse and other addictions. 19 out of 34 individuals who were involved in substance abuse were depressed. However, it was important to note that the depressed individuals resorted more to other means of addictions (62.4%) as opposed to substance abuse.

**Table S2** details the health, lifestyle practices/issues and behavioural issues among the adolescents among the respondents.

A vast majority of them (59.6%) watched movies. Less than half (24.4%) of these expressed a liking for listening to music, with “rock” being the most favourite type of music amongst them. More than half of the respondents (64.8%) had access to the internet, out of which, the majority (273/401) reported to use less than 20 hours per week. More than two-thirds of the adolescents (71.8%) followed trends in fashion. Only (16.8%) of the respondents opined that physical activity is essential for health.

Thirty-five adolescents out of all the respondents were smoking cigarettes currently. A significant majority was aware of the hazards associated with smoking. Smoking among the respondents is detailed in **Table 1**.

**Table 1:** Smoking among respondents (n = 401).

Question/Response		Number	(Percent)
1. Smoke cigarettes presently		35	8.7
2. Started smoking:	<12 years of age	10	28.6
	>12 years of age	25	71.4
3. Smoke(cigarettes/day):	1–5	32	91.4
	6–19	1	2.8
	>20	2	5.6
4. Adverse effects of smoking:	Cancer	224	55.9
	Lung disease	236	58.9
	Heart disease	123	30.7
5. Why started to smoke?	Peer pressure	13	37.1
	Parent smokes	4	11.4
	Get relief from stress	10	28.6
	Gives confidence	3	8.6
	Don't know	5	14.3
6. Intend to stop smoking?	Never	2	5.7
	Not thought about it	14	40
	Yes	19	54.3

Most of the adolescents with lifestyle issues fell in the age group of 16–18 years. Females were more depressed than males and had more sleep problems. Substance abuse and other addictions were documented more in males. **Table 2** elucidates the association of lifestyle practices with age and gender.

**Table 2:** Association of common lifestyle practices/issues with age and gender.

Health, Lifestyle and Behaviour Characteristics	Most common age(in years)/	GenderM(%)/F(%)
Sleep	18	22/78
Depression	18	34/66
Substance abuse	17	85/15
Other addictions	17	92/8

## Discussion

The most commonly reported health and lifestyle risk behaviours among our respondents were inadequate sleep, depression and smoking. The least prevalent health risk behaviors were substance abuse and undereating.

Our results show that (58.9%) of the respondents are getting less than eight hours of sleep daily, which is a cause for concern and further enquirzy [22]. Carskadon, a researcher at brown university discovered other important patterns in adolescent sleep. By studying alertness, she determined that teens, far from needing less sleep, actually needed as much or more sleep than they had gotten as children — nine and a quarter hours (Carskadon 1999). Efforts should be geared towards improving teenagers' sleep patterns through informing youngsters, parents, and physicians about proper sleep hygiene and the risks of poor sleep habits.

More than half of the respondents (61.8%) stayed up late night to watch television or listen to music. A study from Taiwan reports similar results, where staying up late was prevalent in 82.3% of the subjects and the second most prevalent health risk behavior [23]. Evidence from literature reveals a significant association between chronic poor sleep and emotional factors, such as worries, anxiety and depression poor sleep hygiene [24]. Moreover, extensive television viewing during adolescence may contribute to the development of sleep problems by early adulthood [25]. Youth risk behaviour surveillance carried out in the US in 2007 also mentions television watching as a ‘priority health risk behaviour [26]. In reducing the number of hours of television watched, these interventions could also positively affect adolescent obesity [27], emotional problems, and academic achievement [28]. More effective school health programs and other policy and programmatic interventions are needed to address this issue.

WHO's report states that at least 20% of young people will experience some form of mental illness - such as depression, mood disturbances, substance abuse, suicidal behaviours or eating disorders [29]. Our results are consistent with the stated evidence as (17%) of our respondents (17%) were depressed. Promoting mental health, and responding to problems if they arise requires a range of adolescent-friendly health care and counseling services in developing countries like Pakistan.

Dental care including brushing of teeth and preventive dental check-ups are considered part of a healthy lifestyle [29]. The status of dental care among the respondents needs improvement.

Very few (84/401) read books outside their curriculum. Research indicates that there is a strong relationship between leisure reading and school achievement(du Toi 2009). Therefore an understanding of adolescents, their motives and interests are necessary when teachers wish to motivate their learners to read a wide range of material for pleasure.

Another area of concern is the dismal involvement of adolescents in extra curricular activities. (23.7%) Participating in extracurricular activities is associated with both short and long term indicators of positive development including school achievement and educational attainment [30]. Hence there is a strong need to encourage adolescents to devote some time to extra curricular activities every week.

It was heartening to note that more than half of the respondents (52.1%) exercised regularly. However we should strive to create further awareness regarding the importance of exercise and physical activity, since very few of them were of the opinion that exercise is essential for health.

The addictive behaviour most common amongst adolescents is cigarette smoking. Almost all regular smokers take up the habit by the age of 18, and half of the 150 million adolescents who continue to smoke will eventually be killed by tobacco related conditions [31].

However, it was encouraging to note that more than half of the respondents (54.3%) wanted to quit smoking. Smoking among adolescents may also be a marker of many other lifestyle and health concerns [32], [33]. Francis, et al. [34] have reported that adolescents who smoke are at higher risk of psychopathology compared to adolescents who are non-smokers [35]. At the same time, (40%) of the respondents had not yet thought of quitting smoking. This is the population that needs to be targeted in order to keep them from smoking.

The number of respondents who ate out with family was twice the ones who ate out with friends. Evidence from literature indicated that the vast majority of children eat most of their meals with family members. Several observational studies have shown that parental presence at meal time is associated with better dietary habits as well as, decreased frequency of skipping breakfast [36]. Family-based interventions are therefore being regarded as effective means for the prevention of childhood obesity and the promotion of healthy lifestyles [37].

There is evidence that children's decisions to smoke are influenced by family and friends. Our study also supports the fact that peer pressure was the most common reason (37.1%) to start smoking. Having a friend who smokes may be an influence in initiating smoking. Livaudais, et al. [38] have reported that having friends who were smokers at baseline was associated with eventually becoming a smoker among Latino adolescents in the United States. Simmons-Norton [39] has reported on the socialization selection effects among adolescents regarding peer smoking.

(11.4%) of the respondents in our study started to smoke since their parents were smokers. The Cochrane review states that family-based programmes can help prevent smoking by children and adolescents [40]. We as family physicians can counsel the parents regarding the influence that their smoking may have on their children, as Moreover, parental disapproval has also been shown to prevent smoking in teenagers (Davis 2002) and this can be adapted as a useful strategy by parents. The same approach may be used to keep the adolescents away from other forms of tobacco use as well, for e.g. sheesha (12.2%) and tobacco paan (areca nut) (7.5%).

Adolescents form the highest proportion of smokers as evident from our research. Therefore, future declines in adolescent tobacco use in could be enhanced by expanding existing tobacco control programs to include prevention and cessation of the use of cigarettes and sheesha, implementing measures that discourage adolescents who have never smoked from initiating tobacco use, introducing programmes in educational institutions regarding the hazards of smoking, expanding legislation to ban exposure to secondhand smoke in all indoor workplaces, and enacting legislation banning pro-tobacco advertising and sponsorship, bans on tobacco advertising, increasing the price of tobacco products through taxation and creating smoke-free areas at schools, colleges, health facilities and sporting venues.

Alcohol consumption is considered part of an unhealthy lifestyle, but fortunately preference for its consumption was minimal among the respondents (1.5%).

A study from New Zealand reports that areca nut (Betel nut) has been chewed since ancient times, but the habit is discouraged because of its oncogenic, addictive and dysaesthetic properties, in addition to having adverse effects on the mucosa, gums and teeth (Yoganathan 2002). The fact that we have found a high prevalence of betel nut chewing (7%) among the respondents in our study warrants a need for preventive strategies in this area.

This study provides a valuable local perspective with regards to the adolescent lifestyle and behaviour. As young people become adults and move out of their parents' orbit, they can become agents of positive change: they have the dynamism and flexibility, but also the perseverance required to make change from within.

Interventions have been found to be successful for lifestyle modification in the general population [41]. Substantial evidence is available in favor of lifestyle interventions leading to a better health outcome [42].

### Strengths and limitations

This survey forms an important document in this direction because very limited amount of research has been done regarding the assessment of adolescent health and behaviour; our study is the first of its kind to the best of our knowledge.

At the same time, we acknowledge that our study had a few limitations. We used convenience sampling to draw our sample; this method is inferior to probability sampling in its representativeness of the population, and this limits the external validity of the study. However, we made an effort to include respondents from different schools of the city to provide better overall representativeness. The information was acquired via a face-to-face interview which was based on a questionnaire. While this may have led to higher rates of completion of the forms because of interviewer's encouragement for optimum completion, it may also have introduced interviewer's bias in the process of data collection despite all efforts to minimize it. The questionnaire was however thoroughly discussed among the interviewers before data collection to reduce interview bias.

### Conclusion

Inadequate sleep, depression and smoking were the leading unhealthy behaviours among the respondents. Adolescents need to be treated as a distinct segment of our population and we suggest that the families of these children can prove to be a great source to help these children live a health life. The study has attempted to highlight various areas of concern with respect to adolescent lifestyle and behaviorist is suggested that the only method of meeting their needs and at the same time aiming to reduce morbidity in this age group is to foster an atmosphere of patient centredness in dealings with adolescent patients and for further research in this important health gain area.

## Supporting Information

Table S1 Demographic profile of the respondents (n = 401).
(0.05 MB DOC)

Table S2 Adolescent health, lifestyle practices/issues and behavior among respondents (n = 401).
(0.09 MB DOC)
