# Supplementary material for: Adolescent Lifestyle and Behaviour: A Survey from a Developing Country
Source: PLoS One. 2010 Sep 27;5(9):e12914. doi: 10.1371/journal.pone.0012914 (PMC2946339; doi:10.1371/journal.pone.0012914)
Supplement: Table S1 — Demographic profile of the respondents (n = 401). (0.05 MB DOC) [file pone.0012914.s001.doc]

**Table S1: Demographic Profile of the Respondents (n=401)**

| Parameters | Number ( percent ) |
| --- | --- |
| Gender:  Males  Females | 226 (56.4) |
| Male |
| Female | 175 (43.6) |
| Age: | 195  14.39 (6.157) |
| 12-15 years |
| 16-18 years | 206 |
| Mean Age in years  (Standard deviation) | 14.39  (6.157) |
| Marital Status: | 02 (0.5) |
| Married |
| Single | 395(99.5) |
| Ethnic origin: | 130 (32.4) |
| Urdu speaking |
| Sindhi  Punjabi Pathan Balochi | 108 (26.9)  103 (25.7)  41 (10.2)  19 (4.7) |
| Punjabi | 103 (25.7) |
| Pathan | 41 (10.2) |
| Balochi | 19 (4.7) |
|  |  |
| Religion: | 380 (94.7) |
| Islam |
| Christianity | 13 (3.2) |
| Hinduism | 07 (1.7) |
| Others | 01 (0.2) |
