# Supplementary material for: Adolescent Lifestyle and Behaviour: A Survey from a Developing Country
Source: PLoS One. 2010 Sep 27;5(9):e12914. doi: 10.1371/journal.pone.0012914 (PMC2946339; doi:10.1371/journal.pone.0012914)
Supplement: Table S2 — Adolescent health, lifestyle practices/issues and behavior among respondents (n = 401). (0.09 MB DOC) [file pone.0012914.s002.doc]

| 1. Suffer/Suffered from: | 253  81  24  204  51  68  138 | 63  20.2  6.0  50.8  12.7  17.0  34.4  2.7  27.4  69.8  36.9  10.7  52.4  13.7  52.1  34.2  56.8  43.1  58.9      61.8  37.9  23.4  16.2  17    6.7    8.4    17.7      1.0  1.5  12.2  6.7  1.0  6.5  2.2 |
| --- | --- | --- |
| i. Hair loss  ii. Near sightedness  iii. Far sightedness  iv. Dental caries  v. Mal-aligned teeth  vi. Skin problems  vii. Headaches |
| 2. Meals taken daily: | 11  110  280 | 2.7  27.4  69.8 |
| One  Two  Three |
| 3. Eating habits: | 148  43  210 | 36.9  10.7  52.4 |
| Overeat  Under eat  Eat just right |
| 4. Water drinking daily (number of regular size glasses) | 55  209  137 | 13.7  52.1  34.2 |
| <4  5-8  >8 |
| 5. Sleep: | 228  173  236 | 56.8  43.1  58.9 |
| Before mid-night  After mid-night  <Eight hours |
| 6. Late night activities: | 248  152  94 | 61.8  37.9  23.4 |
| Watching television/listening to music  Internet use Studying Socializing/eating out |
| 7. Often feel depressed | 65 | 16.2 |
| 8. Often feel anorexic | 68 | 17 |
| 9. Received treatment for depression | 27 | 6.7 |
| 10. Involved in substance abuse | 34 | 8.4 |
| 11. Indulged in use of:    Sleeping pills  Alcohol  Sheesha  Areca nut chewing  Pan chewing  Naswar (Tobacco) chewing  Others | 71    04  06  49  27  04  26  09 | 17.7  1.0  1.5  12.2  6.7  1.0  6.5  2.2 |

**TABLE S2**

| **Question/Response** | **Number** | **(Percent)** |
| --- | --- | --- |

**Adolescent Health,Lifestyle Practices/Issues and Behavior among respondents (n=401)**

| **Question/Response** | **Number** | **(Percent)** |
| --- | --- | --- |
| 12. Favorite Television channels: | 239  55  205  120  155  134 | 59.6  13.7  51.1  29.9  38.7  33.4  24.4    49.4  18.2  13  8.5  4.2  64.8  68.1  13  6.7  71.8  30.4  15.2  20.9  23.7  16.7  52.1  14.5  37.7  4.5  9.0 |
| Movies  News Channels  Music  Cartoons  Sports  Science Channels |
| 13. Often listen to music | 98 | 24.4 |
| 14. Choice of music: Rock  Pop  Old Music  Classical  Other | 198  73  52  34  17 | 49.4  18.2  13  8.5  4.2 |
| 15. Have access to web | 260 | 64.8 |
| 16. Internet use per week: | 273  52  27 | 68.1  13  6.7 |
| < 20 hours  20-40 hours  >40 hours |
| 17. Follow trends in fashion | 288 | 71.8 |
| 18. Eat out often: | 122  61 | 30.4  15.2 |
| With family  With friends |
| 19. Often read books outside course | 84 | 20.9 |
| 20. Often involved in extra-curricular activities: | 95 | 23.7 |
| 21. Physical exercise: | 67  209  58  151  18  36 | 16.7  52.1  14.5  37.7 |
| Is essential for health  Is regular part of weekly routine  > three times per week  > one hour each time  22. Often bullied by others  23. Often bully other |
| 22. Often bullied by others | 18 | 4.5 |
| 23. Often bully other | 36 | 9.0 |
